# The relationship between urologic cancer outcomes and national Human Development Index: trend in recent years

**DOI:** 10.1186/s12894-022-00953-5

**Published:** 2022-01-10

**Authors:** Xiao-Fang Xia, Yi-Qiu Wang, Shi-Yi Shao, Xin-Yu Zhao, Shi-Geng Zhang, Zhong-Yi Li, Yi-Chu Yuan, Nan Zhang

**Affiliations:** 1 Department of Urology, The Second Affiliated Hospital, Zhejiang University School of Medicine, Hangzhou, 310009 China; 2 Department of Hepatobiliary and Pancreatic Surgery, First Affiliated Hospital, Zhejiang University School of Medicine, Hangzhou, 310003 China; 3 Department of Urology, Renji Hospital, Shanghai Jiao Tong University School of Medicine, Shanghai, 200127 China

**Keywords:** Urologic cancer, Human Development Index, Incidence, Mortality, Survival

## Abstract

**Objectives:**

To describe the influence of the socioeconomic development on worldwide age-standardized incidence and mortality rates, as well as mortality-to-incidence ratio (MIR) and 5-year net survival of urologic cancer patients in recent years.

**Methods:**

The Human Development Index (HDI) values were obtained from the United Nations Development Programme, data on age-standardized incidence/mortality rates of prostate, bladder and kidney cancer were retrieved from the GLOBOCAN database, 5-year net survival was provided by the CONCORD-3 program. We then evaluated the association between incidence/MIR/survival and HDI, with a focus on geographic variability as well as temporal patterns during the last 6 years.

**Results:**

Urologic cancer incidence rates were positively correlated with HDIs, and MIRs were negatively correlated with HDIs. Prostate cancer survival also correlated positively with HDIs, solidly confirming the interrelation among cancer indicators and socioeconomic factors. Most countries experienced incidence decline over the most recent 6 years, and a substantial reduction in MIR was observed. Survival rates of prostate cancer have simultaneously improved.

**Conclusion:**

Development has a prominent influence on urologic cancer outcomes. HDI values are significantly correlated with cancer incidence, MIR and survival rates. HDI values have risen along with increased incidence and improved outcomes of urologic caner in recent years.

**Supplementary Information:**

The online version contains supplementary material available at 10.1186/s12894-022-00953-5.

## Introduction

With the growing and aging population, cancer has been expected to rank as the leading cause of death and the most important barrier to increasing life expectancy across the world [1]. Urologists are the front line for the diagnosis and treatment of urologic malignancies, mainly including prostate, bladder, and kidney cancer [2]. According to Global Cancer Statistics 2018, prostate, bladder and kidney cancers have ranked the 3rd, 10th and 14th most common tumors worldwide with crude incidence rate (per 100,000) of 36.0, 7.4 and 5.5, respectively [1]. The main epidemiologic characteristics of urologic cancer are impacted by the large geographic and temporal variation in risk factors related with behavioral, environmental and socioeconomic reasons [2]. Meanwhile, the global composition of urologic cancer patients has been continuously evolving due to multiple forces [1–9].

Socioeconomic development is closely interconnected with public health [10]. Human Development Index (HDI) is the gold standard for the comparison of socioeconomic development, quantified by the composite measures of health, education, and economy [11]. Multiple studies had demonstrated that cancer outcomes were related with HDI [12, 13]. Some identified there was a negative correlation between standardized mortality rates and HDI [14] but others verified no significant correlation [15]. The impact of national HDI on outcomes of urologic malignancies has not been characterized on a global scale. Furthermore, the different urologic cancer profiles in individual countries signify that marked geographic diversity still exists nowadays, with a persistence of local factors in populations at quite unbalanced phases of social and economic transition. But the global distribution and transition of urologic neoplasms under social development and medical advances in recent years are still not clarified.

This study aims to evaluate the correlation between the HDI values and the incidence rates, mortality-to-incidence ratio (MIR) and 5-year survival rates of urologic cancer in 2012 and 2018.

## Materials and methods

### Data sources

Several databases providing reliable data and resources were adopted in this study. The GLOBOCAN database (http://gco.iarc.fr), maintained by the International Agency for Research on Cancer (IARC), provides high-quality registry data of cancer incidence and mortality at the global level, while the CONCORD-3 program is regarded as the largest and most up-to-date study of international cancer survival trends. The United Nations Development Programme (UNDP) database (http://hdr.undp.org/en/statistics) provides human development indicators across multiple dimensions and for every nation, giving an overview of the state of development worldwide.

The incidence and mortality estimates of urologic cancer were originally extracted from the GLOBOCAN database. Patients with urologic cancer were identified by ICD-10 (International Statistical Classification of Diseases and Related Health Problems-10th Revision codes C00-C97) codes for prostate (C61), bladder (C67), and kidney (C64-65, including renal pelvis). Data within 186 countries in 2018 and 175 countries in 2012 were incorporated. The incidence and mortality data were age-standardized rates (ASRs) per 100,000 person-years. The ASRs were calculated according to the world standard population, allowing comparisons between populations without being influenced by differences in their age structures [1, 16].

HDI data for United Nations members of 2012 and 2018 were publicly available in the UNDP database. Countries then were divided into 4 subgroups according to HDI levels by the UNDP (very-high, high, medium and low HDI) [11].

We further collected the 5-year net survival estimates of patients diagnosed with prostate cancer during the 2005–2009 and 2010–2014 periods from the CONCORD-3 report which corresponded with patient status in year 2012 and 2018 [17]. Net survival is the cumulative probability of surviving up to a given time since diagnosis (e.g., 5 years) after correcting for other causes of death (background mortality) [17]. The net survival estimates were also age-standardized by the International Cancer Survival Standard weights [18].

### Statistical analysis

With the obtained incidence and mortality rates, we calculated the prostate, bladder and kidney cancer mortality-to-incidence ratios (MIR) (i.e., cancer deaths divided by incident cancer cases). Extreme values (0, 1 or > 1) were considered abnormal and were excluded from the analysis. To examine patterns in the MIR of urologic cancer by levels of socioeconomic development, we correlated the MIRs to the corresponding HDIs via linear and nonlinear regression. Linear regression fit was conducted to identify the existence of correlation. Correlation was established with a significant *p* value in the nonparametric Spearman correlation test. Nonlinear regression was based on a modified “dose-to-response” model using the formula ${\text{MIR}} = \frac{1}{{1 + 10^{{{\text{HDI}}_{50} - {\text{HDI}} \times {\text{Slope}}}} }},$ HDI_50_ refers to the half-maximal controlled HDI (equivalent to the HDI value at half-maximal MIR) and slope is a parameter indicating the steepness of the fitted curve. MIRs comparison among 4-tier HDI groups was analyzed via One-way ANOVA followed by Tukey–Kramer post hoc tests. We further examined the correlation of national incidence rates and 5-year net survival estimates with corresponding HDI, separately. In order to determine the effects of socioeconomic transitions on urologic cancer outcomes, we further compared the age-standardized MIR or 5-year net survival estimates in the year of 2012 and 2018 (paired t-test). A *p* value less than 0.05 was considered statistically significant. Statistical analysis and plotting were performed using Prism 7 (GraphPad, San Diego, CA).

Global geographical maps showing the gradient distribution of HDI values, incidence, mortality, calculated MIR, and survival estimates were depicted by TileMill (a GitHub software maintained by MapBox, Washington, DC), with map data sources from the Natural Earth database rendered by the Mapnik Library (https://mapnik.org/).

## Result

### Overview of current global urologic cancer epidemiology

**Fig. 1:**
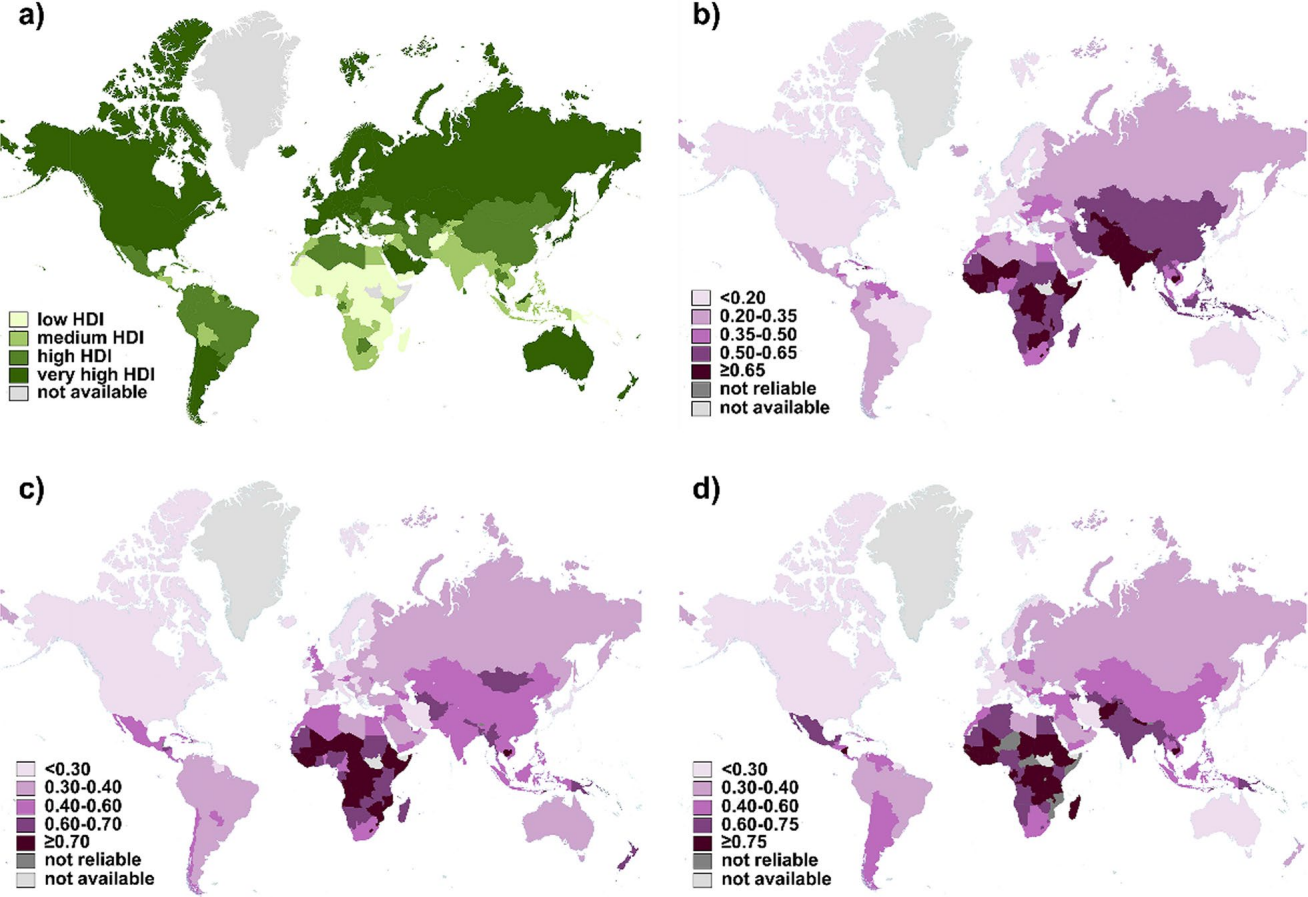
Worldwide distribution of HDI values and MIR of urologic cancer. A total of 174 countries were included into analysis. **a** Countries were classified into 4 tiers according to different levels of HDI (green). **b** Calculated MIR results of prostate cancer, **c** bladder cancer, and **d** kidney cancer were indicated in a purple-gradient color scale. Countries with data unavailable (light gray) or unreliable (dark gray) were denoted. HDI, Human Development Index; MIR, mortality-to-incidence ratio

The data of 174 countries available both in the 2018 GLOBOCAN database and 2018 HDI statistics were incorporated. Development levels of countries were classified into 4 classes according to HDI values by the UNDP (Fig. 1a). The global age-standardized incidence and mortality rates of prostate, bladder and kidney cancer in 2018 were presented separately (**Additional file** 1: Fig. S1a–f). Mortality-to-incidence ratios (MIRs) were calculated for prostate, bladder and kidney cancer and their global distribution was depicted in the form of world maps (Fig. 1b–d). It is estimated that there will be almost 1.8 million new cases of urologic cancer and 616,000 associated deaths worldwide in 2018, showing slightly decreasing tendency compared with 2012 (2.2 million new cases and 734,000 deaths). The global MIR of urologic cancer was 0.282 in 2018 and 0.286 in 2012, with almost no fluctuation.

Prostate cancer had a cumulative 1.3 million new cases and 359,000 deaths worldwide in 2018, ranking as the second most frequent cancer in men. The top countries with the highest incidence rates of prostate cancer were all in very-high HDI group (**Additional file** 1: Fig. S1a), including Europe (e.g., Ireland, Estonia, Norway, Sweden, France, United Kingdom, Czech Republic, Latvia, Slovenia, Luxembourg, Switzerland, Denmark), North America (United States), Australia/ New Zealand, and Barbados. However, mortality rates did not follow those of incidence. The highest mortality rates fell mainly in countries with lower HDI (**Additional file** 1: Fig. S1b), including the Caribbean (e.g., Barbados, Jamaica, Haiti, Saint Lucia, Bahamas, Trinidad and Tobago) and Africa (e.g., Benin, Cabo Verde, Zimbabwe, Liberia, Côte d'Ivoire). The calculated MIRs ranged from 0.081 (France) to 0.844 (Afghanistan). The lowest MIRs were achieved in highly developed countries (Fig. 1b), such as France, Ireland, Italy, Spain, United States, Luxemburg, Israel, Australia, and Japan. Whereas low-to-medium HDI countries owned the highest MIRs (Afghanistan, Guinea, Pakistan, Liberia, Uzbekistan, Cambodia and Nepal).

It was estimated that bladder cancer ranked the 10th most common cancer with 549,000 new cases and 200,000 deaths in 2018. Bladder cancer was most common to happen in high-to-very high HDI regions (**Additional file **1: Fig. S1c), especially European countries (Greece, Denmark, Hungary, Netherlands, Albania, Italy, Germany, Spain), although the highest rates were in Lebanon. However, the top 10 countries with highest MIRs of bladder cancer were all from low-HDI group (Fig. 1c), consisting of Africa (Niger, Comoros, Central African, Djibouti, Liberia, Guinea-Bissau, Guinea, Uganda, Chad) and Timor-Leste. The lowest MIR was 0.119 in Iceland.

403,000 cases of kidney cancer and 175,000 related deaths occurred in 2018. New kidney cancer diagnoses were made most in very-high HDI European countries (Additional file 1: Fig. S1e) (Belarus, Latvia, Lithuania, Czech Republic, Estonia, Slovakia, France, Hungary, Iceland, Croatia). Similarly, the distribution of MIRs of kidney cancer was from 0.165 (Korea) to 0.950 (Mali) (Fig. 1d). The lowest MIRs were achieved in very-high HDI group like Korea, Bahamas, Luxembourg, United States, Canada, Norway, Italy, Australia, and Japan. While the lowest were obtained from low-HDI African countries (Mali, Chad, South Sudan, Burkina Faso, Guinea, Sierra Leone, Eritrea).

### The correlation between urologic cancer MIR and national HDI

**Fig. 2:**
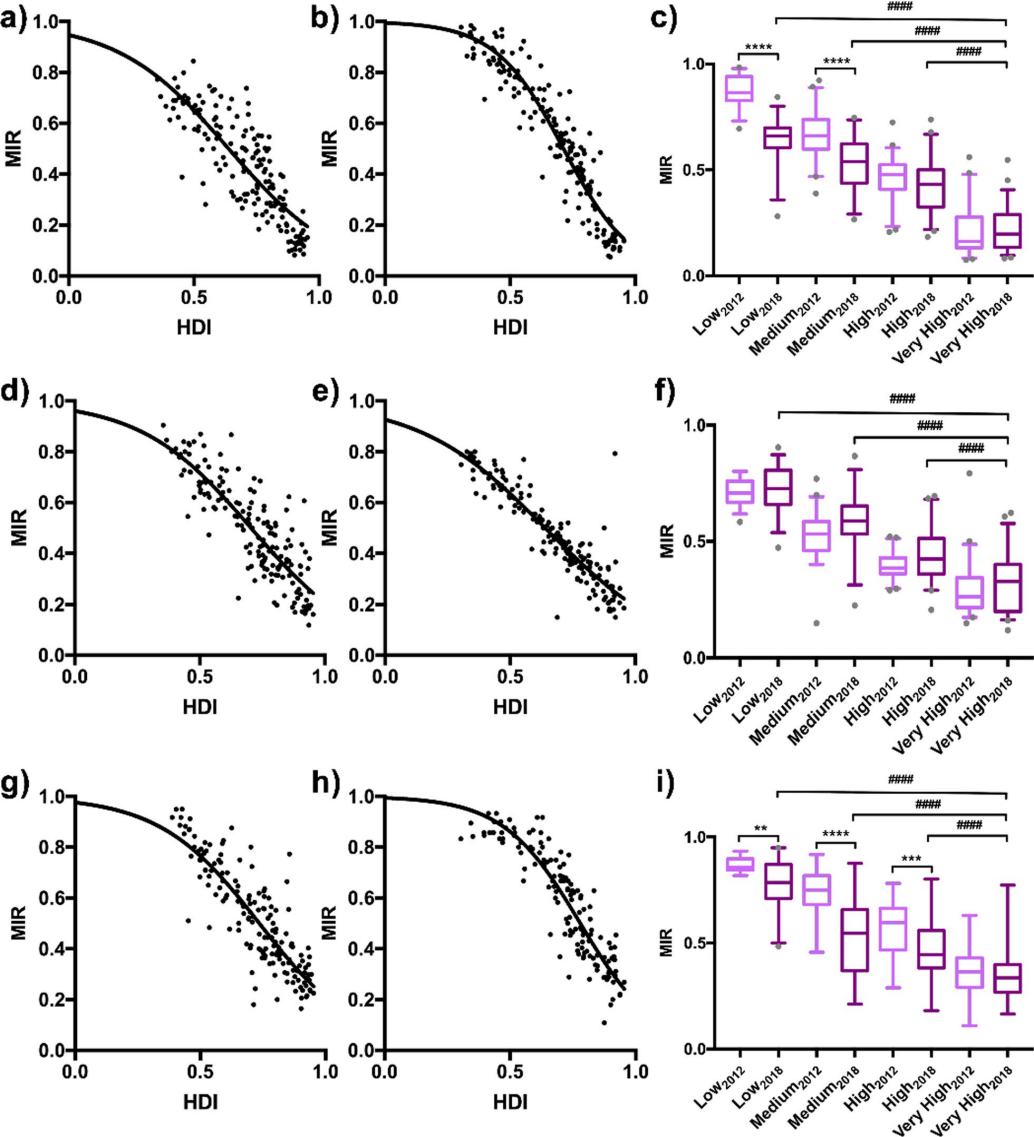
Correlation between HDI and MIR and its transition from 2012 to 2018. The patterns of urologic cancer MIRs to national HDIs with the best-fit lines by modified nonlinear regression (“dose-to-response” model) were presented as following: **a** prostate cancer in 2018 (slope = − 1.962, HDI_50_ = 0.639, R^2^ = 0.687) and **b** in 2012 (slope = − 3.177, HDI_50_ = 0.713, R^2^ = 0.891); **d** bladder cancer in 2018 (slope = − 1.967, HDI_50_ = 0.704, R^2^ = 0.733) and **e** in 2012 (slope = − 1.720, HDI_50_ = 0.640, R^2^ = 0.835); **g** kidney cancer in 2018 (slope = − 2.178, HDI_50_ = 0.736, R^2^ = 0.737) and **h** in 2012 (slope = − 2.835, HDI_50_ = 0.780, R^2^ = 0.824). MIRs of **c** prostate, **f** bladder and **i** kidney cancer in the 4 HDI groups, with significant differences among the very high, high, medium and low groups and a decreasing tendency in certain groups between 2012 (light purple) and 2018 (dark purple). #### *p* < 0.0001, *vs.* very-high-HDI countries in 2018, one-way ANOVA followed by Tukey–Kramer post hoc test. The statistical significance among countries in 2012 was not indicated. **p < 0.01, ***p < 0.001, ****p < 0.0001, 2008 *vs.* 2018 in specific corresponding group, unpaired t-test

The global MIR of prostate, bladder and kidney cancer in 2018 was 0.358, 0.251, and 0.410, respectively (Fig. 1b–d). As the above described distribution of urologic cancer MIR and developing degree, we analyzed their relationship in mathematical regression. We found that as the level of national HDI increased, the corresponding urologic cancer MIR was relatively lower, with strong correlation (prostate: *r* = − 1.059, *p* < 0.0001 for 2018, *r* = − 1.425, *p* < 0.0001 for 2012; bladder: *r* = − 1.049, *p* < 0.0001 for 2018, *r* = − 0.918, *p* < 0.0001 for 2012; kidney: *r* = − 1.153, *p* < 0.0001 for 2018, *r* = − 1.231, *p* < 0.0001 for 2012). We also applied nonlinear regression analysis on data, verifying the existence of a “dose-to-response” inhibitory effect between HDI values and MIRs (Fig. 2a, b, d–e, g, h). The HDI values at half maximal MIR (HDI_50_) of prostate, bladder and kidney cancer in 2018 was 0.639, 0.704 and 0.736, respectively.

We further compared the MIRs of urologic cancer among 4-tier HDI groups and clarified the persistent disparities associated with HDI levels (*p* < 0.0001, One-way ANOVA). Take prostate cancer in 2018 for example, the mean MIR in very-high HDI countries (0.224) was significantly lower than that in high- (0.424), medium- (0.522), or low- (0.641) HDI countries (*p* < 0.0001, Tukey's post hoc test; Fig. 2c). Similar results were obtained in other cancer sites (bladder, kidney) as well as data in 2012 (*p* < 0.0001, Tukey's post hoc test; Fig. 2f, i).

### Association between incidence rates of urologic cancer and HDI

**Fig. 3:**
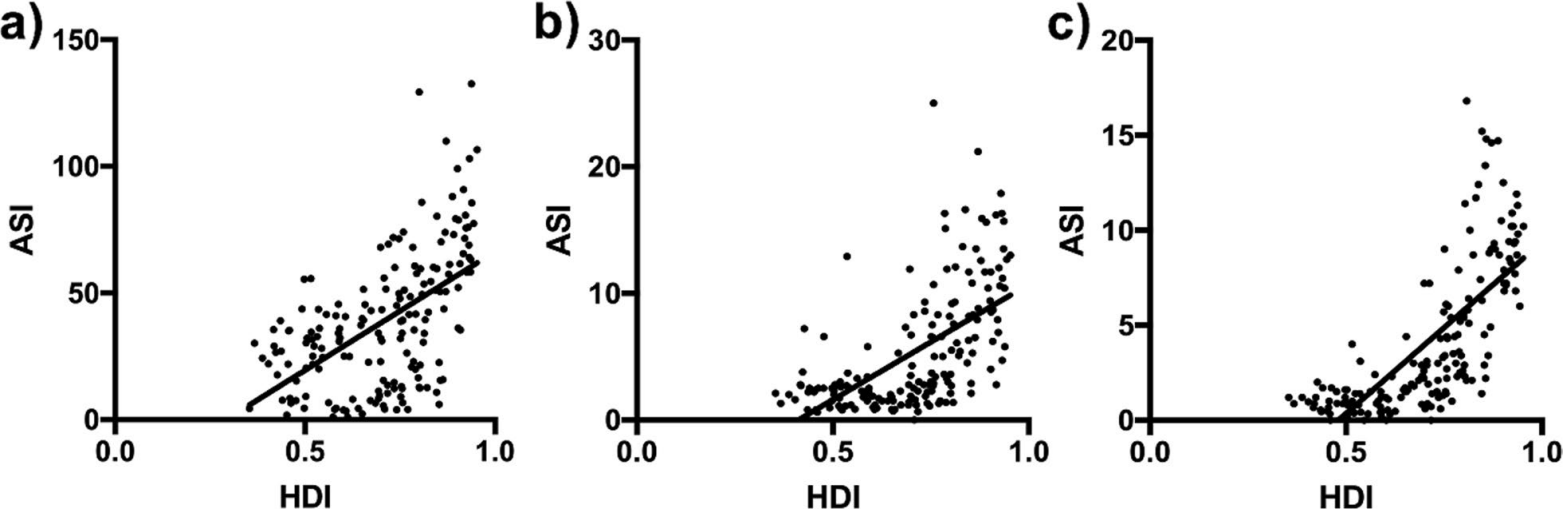
The association between incidence rates of urologic cancers and HDI. **a** The national age-standard incidence rates of prostate cancer correlated positively (r = 0.556, *p* < 0.0001) with HDIs via linear regression (slope = 93.54) in 2018. Similar results for **b** bladder cancer (r = 0.661, *p* < 0.0001, slope = 18.22) and **c** kidney cancer (r = 0.816, *p* < 0.0001, slope = 18.06)

Since the fact that urologic cancers tended to happen more in high-to-very high-HDI countries, we also applied correlation analysis on the association between incidence rates and HDI. It was demonstrated that national incidence rates in urologic cancer all had strong correlation with corresponding HDIs via linear regression (*r* > 0, *p* < 0.0001; Fig. 3a–c).

### The impact of HDI on 5-year survival of prostate cancer

**Fig. 4:**
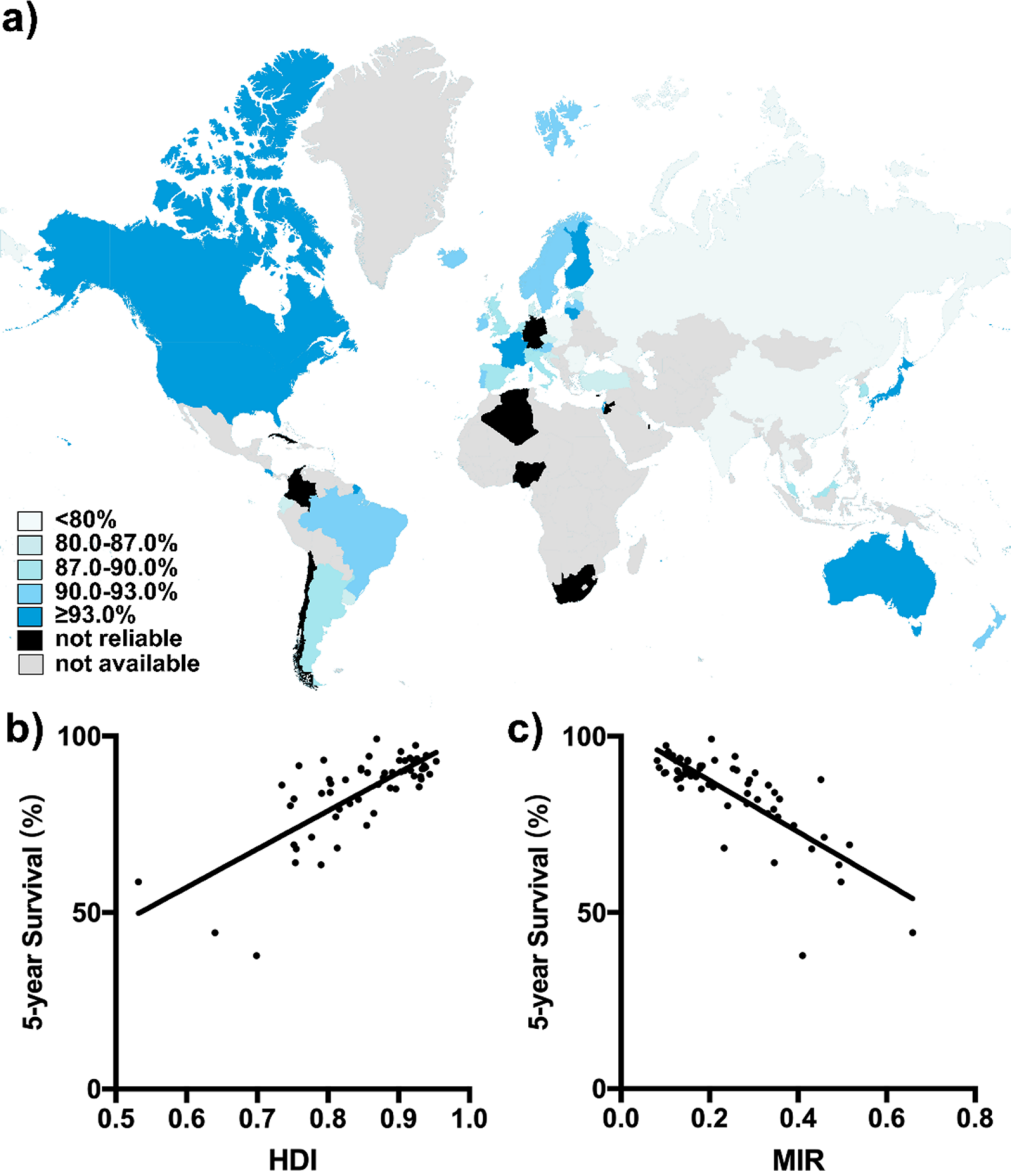
Distributions of prostate cancer survival and its correlation with HDI values and MIRs. **a** Distribution of regional estimated 5-year net survival for patients with prostate cancer in 2018, indicated in blue-gradient colors. **b** A positive correlation pattern between the survival of the patients diagnosed in 2010–2014 and the HDI value in 2018 (*r* = 0.669, *p* < 0.0001, slope = 108.4). **c** Correlation between national MIR and survival of prostate cancer in 2018 (*r* = − 0.749, *p* < 0.0001, slope = − 72.97)

The 5-year net survival rates were available in 57 countries for prostate cancer in CONCORD-3 program (Fig. 4a). Similar to MIRs, patients diagnosed during 2010–2014 from very-high-HDI countries like Cyprus (99.2%), United States (98.1%) and Israel (95.6%) topped in survival rates. While countries with limited developments, like South Africa (37.8%), India (44.3%) and Nigeria (58.7%), fell far behind other regions. We then investigated the association between survival rates and HDI. Cross-national analysis demonstrated that survival rates of patients diagnosed in 2010–2014 correlated positively with HDI values via linear regression (*r* = 1.084, *p* < 0.0001; Fig. 4b). Accordingly, the survival rates correlated inversely with national MIR (*r* = − 0.730, *p* < 0.0001; Fig. 4c).

### Temporal transition of urologic cancer burdens and outcomes from 2012 to 2018

It was reported that urologic cancers had a cumulative 2.1 million new cases worldwide in 2013, nearly 2.5-fold the number in 1990 [2]. Our study updated the transition of urologic cancer outcomes from 2012 to 2018 with latest data. Also, the calculated summary statistics were listed in Table 1.

**Table 1:** Summary statistics of all variables of bladder, prostate and kidney cancer

	2012				2018			
Cancer type	HDI	ASI	MIR	Survival	HDI	ASI	MIR	Survival
prostate	0.669/0.702 (0.519–0.805)	41.0/35.4 (16.2–58.3)	0.542/0.543 (0.338–0.777)	82.2/87.3 (76.6–91.1)	0.705/0.735 (0.582–0.826)	38.7/35.2 (15.8–55.8)	0.432/0.434 (0.276–0.606)	84.1/88.2 (80.6–91.4)
bladder		5.32/3.2 (1.90–7.95)	0.473/0.444 (0.347–0.631)	NA		5.31/3.00 (1.90–8.13)	0.497/0.497 (0.344–0.643)	NA
kidney		4.05/2.60 (1.20–6.05)	0.582/0.604 (0.386–0.780)	NA		4.04/2.30 (1.19–6.43)	0.518/0.492 (0.360–0.684)	NA

The statistics are presented as mean/median (inter quartile range). HDI, Human Development Index. ASI: Age-Standardized Incidence. MIR, Mortality-to-Incidence Ratio

#### Prostate cancer

**Fig. 5:**
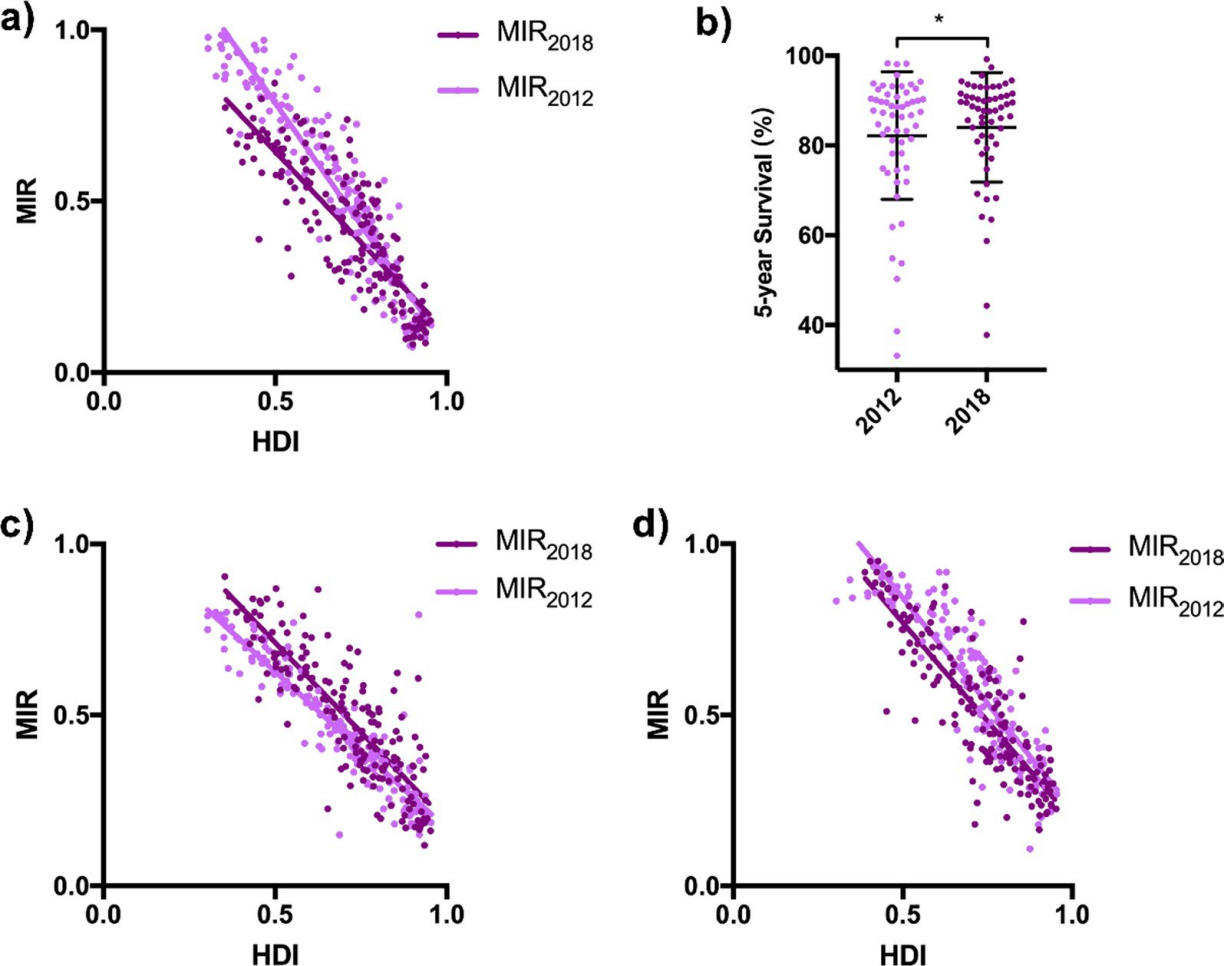
Urologic cancer outcomes and its trend from 2012 to 2018. Transition of the correlation patterns of **a** prostate cancer MIR to national HDI from 2012 (light purple, r = − 0.617, *p* < 0.0001) to 2018 (dark purple, r = − 0.548, *p* < 0.0001), **c** bladder cancer from 2012 to 2018, as well as **d** kidney cancer from 2012 to 2018, showing a declining tendency of MIRs within the decade. **b** Significant increase in overall survival rates in 57 overlapping countries from 2012 (light purple) to 2018 (dark purple). *p < 0.05, paired t-test

Incidence and mortality rates of prostate cancer have risen considerably since the end of last century [2]. Nevertheless, it should be noted that between 2012 and 2018, new prostate cases decreased from 1,276,706 to 1,111,689, and fell from 358,989 to 307,417 in deaths. In general, the MIR of prostate cancer did not change much during the past 6 years (0.251 *vs* 0.259). We plotted national HDIs and MIRs in 2018 and 2012 together and noticed similar distributions (Fig. 5a). Notably, there is an evident change that linear regression line has shifted to the lower-left direction from 2012 to 2018, most obviously among countries with lower HDIs. Furthermore, within both low- and medium- HDI groups, the national MIRs in 2018 decreased significantly in comparison to the 2012 data (*p* < 0.0001 for both groups, Mann–Whitney test; Fig. 2c). In high HDI groups, there was only decreasing tendency without significance (0.424 *vs* 0.460, *p* > 0.05; Fig. 2c). The survival rates generally increased, with only 7 out of 57 countries dropped more than 1% (*p* < 0.05; paired t-test) (Fig. 5b; Table 2).

**Table 2:** National HDI and 5-year net survival values of prostate cancer from 2012 to 2018

Country	2012		2018		Transitions in a decade	
	HDI	Survival	HDI	Survival	Δ_HDI_	Δ_survival_
Algeria	0.713	50.3	0.754	64.1	0.041	13.8
Argentina	0.811	83.6	0.825	87.6	0.014	4
Australia	0.938	93.2	0.939	94.5	0.001	1.3
Austria	0.895	90.8	0.908	90.2	0.013	− 0.6
Belgium	0.897	93.2	0.916	93.8	0.019	0.6
Brazil	0.73	92.5	0.759	91.6	0.029	− 0.9
Bulgaria	0.782	54.8	0.813	68.3	0.031	13.5
Canada	0.911	94.2	0.926	93.6	0.015	− 0.6
Chile	0.819	84.4	0.843	82	0.024	− 2.4
China	0.699	62.5	0.752	69.2	0.053	6.7
Colombia	0.719	87.8	0.747	80.3	0.028	− 7.5
Costa Rica	0.773	92.6	0.794	93.2	0.021	0.6
Croatia	0.805	78.3	0.831	80.9	0.026	2.6
Cuba	0.78	53.8	0.777	71.4	− 0.003	17.6
Cyprus	0.848	98.3	0.869	99.2	0.021	0.9
Czech Republic	0.873	81.5	0.888	85.3	0.015	3.8
Denmark	0.901	82.5	0.929	85.6	0.028	3.1
Ecuador	0.724	80.7	0.752	82.2	0.028	1.5
Estonia	0.846	83.2	0.871	86.3	0.025	3.1
Finland	0.892	93.4	0.920	93.2	0.028	− 0.2
France	0.893	93.6	0.901	93.1	0.008	− 0.5
Germany	0.92	91.8	0.936	91.6	0.016	− 0.2
Iceland	0.906	89.7	0.935	90.8	0.029	1.1
India	0.554	33.2	0.640	44.3	0.086	11.1
Ireland	0.916	89.7	0.938	91.1	0.022	1.4
Israel	0.9	95.7	0.903	95.6	0.003	− 0.1
Italy	0.881	89.6	0.880	89.5	− 0.001	− 0.1
Japan	0.912	91.4	0.909	93	− 0.003	1.6
Jordan	0.7	88.6	0.735	86.1	0.035	− 2.5
Korea, Republic of	0.909	87.3	0.903	89.9	− 0.006	2.6
Kuwait	0.79	71.9	0.803	84	0.013	12.1
Latvia	0.814	88.8	0.847	90.4	0.033	1.6
Lithuania	0.818	93.8	0.858	94.3	0.040	0.5
Malaysia	0.769	74.9	0.802	87.7	0.033	12.8
Malta	0.847	86.4	0.878	88.2	0.031	1.8
Mauritius	0.737	61.8	0.790	63.5	0.053	1.7
Netherlands	0.921	87.5	0.931	88.5	0.010	1
New Zealand	0.919	89.3	0.917	90.3	− 0.002	1
Nigeria	0.471	73.9	0.532	58.7	0.061	− 15.2
Norway	0.955	90.3	0.953	92.9	− 0.002	2.6
Poland	0.821	75	0.865	78.1	0.044	3.1
Portugal	0.816	90	0.847	90.9	0.031	0.9
Qatar	0.834	98.2	0.856	89.6	0.022	− 8.6
Romania	0.786	78.2	0.811	77.1	0.025	− 1.1
Russian Federation	0.788	68.6	0.816	79.3	0.028	10.7
Singapore	0.895	86.7	0.932	87.8	0.037	1.1
Slovakia	0.84	74.4	0.855	74.7	0.015	0.3
Slovenia	0.892	83.2	0.896	85	0.004	1.8
South Africa	0.629	38.6	0.699	37.8	0.070	− 0.8
Spain	0.885	90.4	0.891	89.7	0.006	− 0.7
Sweden	0.916	90.1	0.933	90.7	0.017	0.6
Switzerland	0.913	88.6	0.944	89.2	0.031	0.6
Thailand	0.69	71.8	0.755	68	0.065	− 3.8
Turkey	0.722	81.2	0.791	83.8	0.069	2.6
United Kingdom	0.875	86.7	0.922	88.7	0.047	2
United States of America	0.937	98.1	0.924	97.4	− 0.013	− 0.7
Uruguay	0.792	84.7	0.804	86.5	0.012	1.8

A total of 57 countries with survival rates available in both years; HDI, Human Development Index

#### Bladder cancer

New bladder cancer diagnoses shrank nearly a quarter between 2012 and 2018 (549 393 *vs* 429 793). It is remarkable that MIR of bladder cancer in 2018 had a slight increase when compared with 2012, though not significantly (0.358 *vs* 0.333; *p* > 0.05, Fig. 2f, Fig. 5c). There was also only tiny fluctuation between MIR of 2012 and 2018 within each specific HDI group (Fig. 2f).

#### Kidney cancer

The incidence of kidney cancer also reduced during 2012–2018 (337 860 *vs* 403 262). In the scatter diagram of HDI-MIR, current MIR of kidney cancer also shifted in the direction of lower-left when comparing with 2012 (Fig. 5d). Remarkably, two regression lines were nearly parallel (r_2018_ =  −  1.153, r_2012_ = − 1.231; *p* < 0.01, Mann–Whitney test). Meanwhile, MIRs of kidney cancer declined overall, across all development status (*p* < 0.01 for the low-, *p* < 0.0001 for the medium-, *p* < 0.001 for the high-, and *p* = 0.23 for the very-high-HDI group, Mann–Whitney test; Fig. 2i).

## Discussion

Our study aims to clarify the latest epidemiology of urologic cancer and the contribution of national development to urologic cancer outcomes. We adopted Human Development Index (HDI) to evaluate development, a composite index focusing on three basic dimensions of socioeconomic development: life expectancy, years of schooling, and gross national income per capita [11]. Mortality-to-incidence ratio (MIR) and 5-year net survival both represent cancer outcomes. MIR is regarded as a quite useful surrogate indicator of oncology care effectiveness, which could be a comprehensive result of screening, diagnostic modality, treatment and follow-up [12, 19]. Meanwhile, 5-year net survival might be labeled with more importance, since cancer patients who survive for a considerable time span can, in a way, be considered cured [17, 20]. In the current study, we proved that all three urologic cancers MIR negatively, while incidence and survival rates positively correlated with HDI. Our study investigated the trends in the global burden of urologic cancer from 2012 to 2018. HDI values raised along with the decline in corresponding incidence and MIRs, as well as improvement in survival.

### Prostate cancer

Incidence of prostate cancer was highest in countries with very-high HDI, like Europe, North America, Australia/New Zealand, as well as Barbados and Bahamas. The public recommendation and prevalence of early diagnostics for prostate cancer in more developed countries, by PSA testing and detection of latent cancer in transurethral prostatectomy or puncture biopsy, led to higher incidence rates. For example, the commercial availability of PSA testing from 1980s brought about the intensively use of the test and rapid growth in new cases, first in the United States and within a few years, in Europe, Australia/New Zealand, and Canada [1, 21, 22]. Another explanation could be attributed to age. Nearly 75% of new prostate cancer cases occurring in people aged over 85 years, and incidence of prostate cancer is directly correlated with age [15, 23]. Since life expectancy is one of key elements of HDI, there is no doubt that countries with higher HDI had a greater prostate cancer incidence. Moreover, ethnic and genetic predisposition could also be blamed for prostate cancer morbidity. The rates are highest among men of African descent in the United States and the Caribbean [24]. That’s why Barbados and Bahama topped in the incidence rates of prostate cancer with a relatively lower HDI within high-HDI group.

Based on previous study, we demonstrated an inverse correlation of MIR and a positive association between survival and HDI. The non-linear regression analysis confirmed the “dose-to-response” effect between HDI and MIR. The relationship between HDI and MIR bears a similarity to the dose-dependent inhibitory response by drugs because of the existence of several characteristics in common, like (1) MIR or response approaches 1 as HDI or dose approaches 0; (2) MIR or response decreases as HDI or dose increases; and (3) MIR or response approaches 0 as HDI or dose approaches infinity. The impact of HDI on cancer outcomes seemed to be driven by national inequalities in health care, resulting in deviations in treatment effectiveness. First of all, the widespread access to diagnostic services and screening tests in more developed countries leads to increased diagnosis at earlier stages of disease and better clinical outcomes. However, we could not deny the overdiagnosis associated with PSA screening, which might also help to decrease mortality rates of individuals identified with prostate cancer. Some new biomarkers sparing those who overdiagnosed are under development (e.g., *PCA3* or *TMPRSS-ERG* fusions) [25]. Secondly, the delivery of urologic oncology care is susceptible to regional variation. Access to effective radiation equipment and neoadjuvant hormonal therapy is linked to a country’s wealth. Globally, there is a mismatch of radiation treatment resources to need, with nearly 4,000 radiation units in the United States and fewer than 300 in sub-Saharan Africa, a region with more than twice the population [26]; up to 36 countries worldwide have even no radiation capabilities [27]. Advances in immunotherapy and robotic surgery, though promising, are not feasible or affordable for generalized application in settings with limited health care resources [28–30]. Individuals in countries with higher HDI possessed heightened awareness and more positive preventative measures against prostate cancer such as smoking cessation and healthy diet.

Multiple studies have reported that the detected cases of prostate cancer from 1990 to 2010 were increasing rapidly, which could also be attributed to the era of PSA testing and ultrasonography, as well as aging population [2, 31]. However, incidence of prostate cancer was on its downhill from 2012 to 2018. The 2012 recommendation against the routine use of PSA testing by the US Preventive Services Task Force (USPSTF) may have partly driven trends downward [32]. American Urological Association (AUA) stated in 2013 update that routine screening of men aged 40–54 years and men with less than a 10- to 15- year life expectancy was not recommended [33]. Meanwhile, our analysis revealed that from 2012 to 2018, the integral worldwide HDI values increased, along with the decline in MIRs and improvement in survival of prostate cancer. Scientific advances have resulted in rapidly growing medical technology and treatment strategies. The development of laparoscopic and robotic surgery, especially the da Vinci Surgical System (Intuitive Surgical, Sunnyvale, CA), has offered a less invasive approach while ensuring oncological remission and expected survival [28]. Meanwhile, novel approaches like immune checkpoint blockades targeting programmed cell death protein 1 (PD-1) or its ligand PD-L1 have also emerged as powerful methods against tumor progression, relapse, and metastasis [29, 30].

### Bladder cancer

Higher incidence rates of bladder cancer could be observed in more developed countries, especially in Southern and Western Europe. The rates were positively correlated with HDI (*p* < 0.0001). Chemical or environmental exposures are major risk factors for bladder cancer, including smoking, obesity, alcohol drinking and red-meat consumption [34, 35, 37]. These risk factors have been reported by the World Health Organization as alarmingly high across Europe [36, 38, 39]. Other likely reason is widespread practice of initial assessment in more developed regions [35, 37]. However, mortality rates of bladder cancers did not follow those of incidence. MIR of bladder cancer appeared to be high in less developed regions. The correlation between MIR and HDI was moderately strong (*p* < 0.0001). Higher-quality medical care and better health awareness were possessed in highly developed areas, as stated in *Prostate Cancer*.

From 2012 to 2018, patients went in declines in both incidence and MIR of bladder cancer worldwide. Primary prevention of tobacco use is the most effective strategy for bladder cancer prophylaxis [23, 40]. Thus, decreasing incidence and earlier diagnosis, improved endoscopic system for cystoscopic surveillance [41], robotic surgery with less invasive injury [42], better intravesical therapy, such as the Bacillus Calmette-Guerin (BCG) and updated chemotherapy [34] and targeting therapy all contributed to bladder cancer outcomes improvement [43].

### Kidney cancer

The incidence of kidney cancer was the highest in very high HDI countries, and correlated positively with country-specific HDI (r = 0.816, *p* < 0.0001). Similarly, higher prevalence of risk factors such as obesity, hypertension, diabetes and smoking played a role in the increased incidence of kidney cancer in developed countries [44–50]. Furthermore, the frequency and quality of cross-sectional imaging tend to be higher in developed nations [23]. Similarly, MIR of kidney cancer negatively correlated with certain national HDI [50].

The global burden and MIR of kidney cancer stagnated or decreased in the majority of countries examined in this study from 2012 to 2018. As discussed, more developed preventative efforts and treating methods could lead to a decrease in cancer incidence and mortality.

There are some limitations to our study. First, cancer registration in relatively less-developed nations could suffer from higher chance of under-reporting due to limited communication infrastructure and less robust recording system; low income and lower willingness to utilize healthcare services; relative lack of clinical services and investigation tests. GLOBOCAN and CONCORD-3 often extrapolates data for certain developing nations based on data from subnational areas or major cities. Second, discrepancies between the reliability of incidence and mortality reporting limit MIR interpretation, as mortality data are generally more accurate than incidence. Although we exclude extreme values (0, 1, or > 1) of MIR, there is no way to correct this bias in our analysis. Third, we could not establish cause-and-effect relationships in correlational analysis.

## Conclusion

In conclusion, HDI values are significantly correlated with urologic cancer incidence, MIRs and survival rates. More developed countries are more likely to have higher incidence and mortality rates, but lower MIRs. From 2012 to 2018, new cases of urologic cancer have declined, with apparent improvement in clinical outcomes. However, we should not relax our vigilance since patients with urological cancer were older and more medically complex, and had more frequent health system contact [51], especially in developing countries where gains in life expectancy and screening prevalence are greater. Disparities in cancer health care should compel us to exert greater effort in improving awareness, universal health coverage, access to either publicly funded or affordable screening programs and treatment in low HDI countries.

## Supplementary Information

**Additional file 1. Fig. S1.** Worldwide distribution of Urologic cancer burden in 2018. A total of 174 countries were included. (**a**), (**c**), (**e**) Age-standardized incidence (blue) and (**b**), (**d**), (**f**) age-standardized mortality (red) rates per 100,000 population of prostate cancer, bladder cancer, and kidney cancer, separately, indicated in a gradient color scale. Countries with data unavailable (light gray) were denoted.

## Data Availability

The incidence and mortality estimates of urologic cancer were originally extracted from the GLOBOCAN database (http://gco.iarc.fr) maintained by the International Agency for Research on Cancer (IARC). The public access to the database is open, and raw data would be available by contacting Dr. Zhang (nanzhang@zju.edu.cn).
