# Supplementary material for: The relationship between urologic cancer outcomes and national Human Development Index: trend in recent years
Source: BMC Urol. 2022 Jan 10;22:2. doi: 10.1186/s12894-022-00953-5 (PMC8744298; doi:10.1186/s12894-022-00953-5)
Supplement: Supplementary file 1 — Additional file 1. Fig. S1. Worldwide distribution of Urologic cancer burden in 2018. A total of 174 countries were included. (a), (c), (e) Age-standardized incidence (blue) and (b), (d), (f) age-standardized mortality (red) rates per 100,000 population of prostate cancer, bladder cancer, and kidney cancer, separately, indicated in a gradient color scale. Countries with data unavailable (light gray) were denoted. [file 12894_2022_953_MOESM1_ESM.docx]

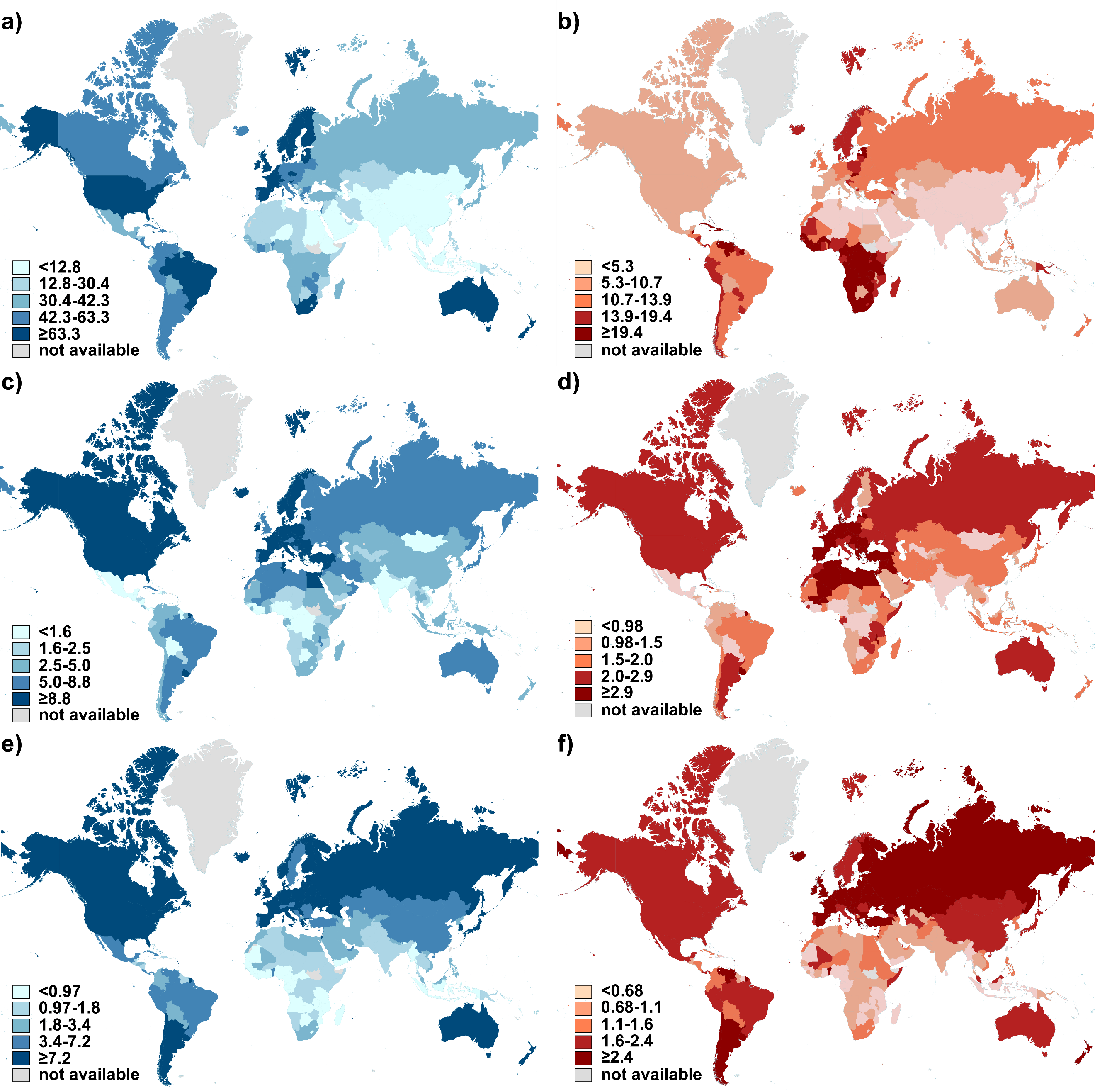


**Fig. S1. Worldwide distribution of Urologic cancer burden in 2018.**

A total of 174 countries were included. (a)(c)(e) Age-standardized incidence (blue) and (b)(d)(f) age-standardized mortality (red) rates per 100,000 population of prostate cancer, bladder cancer, and kidney cancer, separately, indicated in a gradient color scale. Countries with data unavailable (light gray) were denoted.
